# 

**Published:** 1893-07-08

**Authors:** 


					PRIOP T\A/nDCKIOC - WW WITH EXTRA NURSING SUPPLEMENT.
No 3^4. Vfti VM/ i ^ i^i 1 onn jl i[ J > I Per Annum, 8s.60., port free.
ra^, "Ol. XIV.?July 8th, 1893. \l^ JBwMl JmjQ Monthly Parts, 6d.? post free, 8d.
AbSST'?' ^ Ditto par Annum, 8.., port free.
^  ??
fITH EXTRA NURSING SUPPLEMENT. ^
Vol. XIV. Saturday,
Jtoy 8th, 1893.
[Twopence.

				

